# Pumpless extracorporeal interventional lung assist in patients with acute respiratory distress syndrome: a prospective pilot study

**DOI:** 10.1186/cc7703

**Published:** 2009-01-30

**Authors:** Markus Zimmermann, Thomas Bein, Matthias Arlt, Alois Philipp, Leopold Rupprecht, Thomas Mueller, Matthias Lubnow, Bernhard M Graf, Hans J Schlitt

**Affiliations:** 1 Department of Anesthesiology, University of Regensburg Medical Center, Franz-Josef-Strauß-Allee 11, Regensburg, 93053, Germany; 2 Department of Cardiothoracic and Vascular Surgery, University of Regensburg Medical Center, Franz-Josef-Strauß-Allee 11, Regensburg, 93053, Germany; 3 Department of Internal Medicine II, University of Regensburg Medical Center, Franz-Josef-Strauß-Allee 11, Regensburg, 93053, Germany; 4 Department of General Surgery, University of Regensburg Medical Center, Franz-Josef-Strauß-Allee 11, Regensburg, 93053, Germany

## Abstract

**Introduction:**

Pumpless interventional lung assist (iLA) is used in patients with acute respiratory distress syndrome (ARDS) aimed at improving extracorporeal gas exchange with a membrane integrated in a passive arteriovenous shunt. In previous studies, feasibility and safety of the iLA system was demonstrated, but no survival benefit was observed. In the present pilot study we tested the hypothesis that timely initiation of iLA using clear algorithms and an improved cannulation technique will positively influence complication rates and management of lung protective ventilation.

**Methods:**

iLA was implemented in 51 patients from multiple aetiologies meeting ARDS-criteria (American-European Consensus) for more than 12 hours. Initiation of iLA followed an algorithm for screening, careful evaluation and insertion technique. Patients with cardiac insufficiency or severe peripheral vascular disease were not considered suitable for iLA. Arterial and venous cannulae were inserted using a new strategy (ultrasound evaluation of vessels by an experienced team, using cannulae of reduced diameter). The incidence of complications and the effects on tidal volumes and inspiratory plateau pressures were primary outcome parameters, while oxygenation improvement and carbon dioxide removal capabilities were secondary study parameters.

**Results:**

Initiation of iLA resulted in a marked removal in arterial carbon dioxide allowing a rapid reduction in tidal volume (≤ 6 ml/kg) and inspiratory plateau pressure. Adverse events occurred in 6 patients (11.9%). The hospital mortality rate was 49%.

**Conclusions:**

The use of an indication algorithm for iLA in early ARDS, combined with a refined application technique was associated with efficient carbon dioxide removal and a reduced incidence of adverse events. iLA could serve as an extracorporeal assist to support mechanical ventilation by enabling low tidal volume and a reduced inspiratory plateau pressure.

## Introduction

Pumpless extracorporeal interventional lung assist (iLA; Novalung, Talheim, Germany) has been described in patients with life-threatening forms of respiratory failure or acute respiratory distress syndrome (ARDS) suffering from persistent hypoxaemia and/or hypercapnia, unresponsive to conventional therapy [1,2]. The iLA system is characterised by a novel membrane gas exchange device with optimised blood flow integrated into an arteriovenous heparin-coated bypass, established by cannulation of the femoral artery and vein. A passive shunt flow generated by the patient's blood pressure gradient through the gas exchange device allows effective carbon dioxide extraction and moderate improvement in arterial oxygenation [3].

We previously examined iLA implementation in a heterogeneous group of 90 severe critically ill patients at our institution and reported a hospital mortality of 59% with a complication rate of 24.4% [4]. Specifically, the patient groups with the highest mortality were identified as patients with cancer, a high demand for vasopressors, advanced age, morbid obesity or those requiring long duration of mechanical ventilation. The most frequent complications reported were related to the arterial cannulation of the femoral vessels, resulting in distal limb ischaemia, compartimental syndrome [4] or bleeding from the cannulation sites requiring surgical intervention [1,5,6].

To date, solid evidence-based outcome data for iLA does not exist, in part, because of inherent patient heterogeneity and the rescue nature associated with the therapy, thereby leaving most iLA protocols to be determined individually 'per case'. An experience-based algorithm-guided approach to implement arteriovenous iLA in the treatment of ARDS has recently been published, setting new criteria for care [7]. The unmet need of clinicians for systematically attained, well-structured studies to reliably define indications for iLA sets a high value on publications of this nature and underscores the importance of establishing proven algorithms for ARDS therapy.

The present prospective pilot study defines an algorithm for cannulation and implementation of iLA while refining inclusion and exclusion criteria because we found evidence that arterial insertion of cannulae with lower diameter might reduce the rate of ischaemic complications. In contrast, the hypothesis that timely initiation of iLA in an early phase of severe respiratory failure, and a consequent tidal volume reduction during iLA therapy will positively influence outcome, will be determined in future randomised controlled trials.

## Materials and methods

### Patients

**Figure 1:**
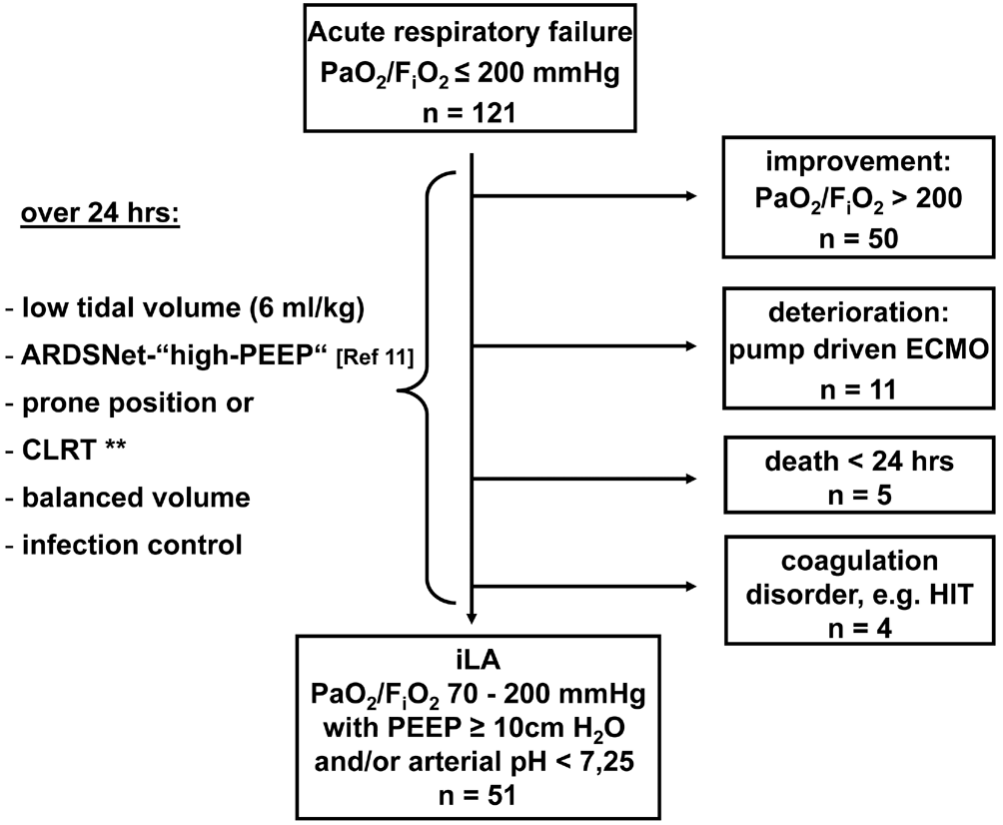
Algorithm for screening, evaluation and implementation of interventional lung assist (iLA). **CLRT = continuous lateral rotation therapy. ARDS = acute respiratory distress syndrome; ECMO = extracorporeal membrane oxygenation; F_i_O_2 _= fraction of inspired oxygen; HIT = heparin-induced thrombocytopenia; PaO_2 _= partial pressure of oxygen in arterial blood; PEEP = positive end expiratory pressure.

After a waiver by the Institutional Review Board at the University of Regensburg Medical Center was granted we report on clinical results, mortality and complication rates in a 'second' prospective cohort of ARDS patients treated by iLA-insertion following an algorithm in comparison to our previously published 'historical' retrospective study [4]. Obtainment of informed consent was deemed by the Institutional Review Board not to be required. Between October 2004 and March 2008, all 121 patients presenting with acute respiratory failure were enrolled in the prospective study. iLA was implemented in 51 patients suffering from ARDS mostly due to pneumonia, trauma or sepsis. The algorithm for screening, evaluation and implementation of interventional lung assist is demonstrated in Figure 1.

Before patients were considered potential candidates for iLA, their clinical course was carefully evaluated. This included optimisation of volume and vasoactive agent requirements, ventilation strategy based on the concept of lung-protective ventilation (moderate hypercapnia, increase in respiratory rate, high positive end expiratory pressure (PEEP)), the use of adjunctive therapeutic measures, for example, prone position [8] or continuous lateral rotation therapy [9], as well as treatment of the underlying disease in accordance to standard intensive care procedures.

After a stabilisation period of 12 to 24 hours (Figure 1), a persisting impairment in pulmonary gas exchange (partial pressure of oxygen in arterial blood (PaO_2_)/fraction of inspired oxygen (F_i_O_2_) 70 to 200 mmHg with PEEP of 10 cmH_2_O or more and/or arterial pH less than 7.25 because of respiratory acidosis [10]) was considered mandatory for the implementation of iLA. In more severe cases of hypoxaemia (PaO_2_/F_i_O_2 _less than 70 mmHg), a pump-driven veno-venous extracorporeal membrane oxygenation (ECMO) was preferably initiated. Patients showing clinical signs of cardiac insufficiency, having severe peripheral vascular disease, those in need of continuously highly dosed vasoactive (noradrenaline greater than 0.4 μg/kg/minute) or inotropic agents were not considered suitable for iLA, because the absence of shock or severe cardiovascular instability is mandatory for the use of iLA.

### Technique

Technical data of the iLA-system has been described in detail previously [3,4]. In principle, iLA is a single use, ultracompact extrapulmonary gas exchange system perfused by the heart. Although carbon dioxide extraction mainly depends on a sufficient sweep gas flow (about 10 L/minute) through the system, patient oxygenation predominantly depends on arteriovenous shunt volume, most strongly influenced by cannulae diameter, and to a lesser extent, by the patients' mean arterial blood pressure.

After ultrasonographic identification and assessment of the diameters of the femoral artery as well as of the contralateral femoral vein, cannulae were implanted by experienced physicians using Seldinger's technique. The size of the arterial cannula was individually selected, based on vessel diameter to ensure sufficient peripheral blood flow with a residual lumen of 30% after insertion. The diameter of the arterial cannula was chosen to be 15 or 17 French (Fr), based conditionally on adequate residual volume. The venous cannula was typically selected two Fr sizes larger in order not to compromise flow resistance. A platelet count or more than 60.000/μl and a partial thromboplastin time less than 60 seconds were primary coagulation needs for implementation of the cannulae.

Basic monitoring of the lower extremities included continuous limb pulse oxymetry distal to the arterial cannulation site, determination of serum lactate and creatine kinase levels as well as clinical inspection for any signs of restricted perfusion and/or ischaemia. An overview of the implementation and monitoring concept is given in Table 1.

**Table 1:** The concept of evaluation, insertion and clinical monitoring of the pumpless interventional lung assist (iLA) in patients with acute respiratory distress syndrome (ARDS)

**Evaluation and preparation**	**Insertion**	**Monitoring**
**Echocardiography**: exclusion of significant cardiac dysfunction	**Preparation** of iLA system and introducer kit	**System**: - continuous calculation of blood flow through the device by transit time Doppler technology
**Ultrasound**: assessment of femoral artery and vein diameter	**Vascular cannula**: **Artery**: allowing a residual lumen ≥ 30% of the vessel diameter maximum 17 Fr (adults)	**Patient**: - continuous limb pulse oxymetry distal the arterial cannulation site (toe)
**Coagulation**: platelets > 60.000/μl aPTT < 60 seconds haemoglobin ≥ 9 mg/dl access to blood bank	**Vein**: + 2 Fr. compared with arterial cannula	- clinical inspection for any signs of restricted perfusion
**Contraindication**: - coagulation disorder e.g. HIT	- cannulation by two experienced physicians	- assessment of serum creatine kinase and lactate regularly
- severe peripheral vascular disease - continuously highly dosed vasoactive or inotropic agents (Noradrenaline > 0.4 μg/kg/minute)	- bolus application of 5000 IU heparin iv	**Arterial blood gases**: - early period (24 hours): frequently = every 4 hours
	- connection of the system stepwise increase of sweep gas flow to 10 l O_2_/minute	- late period (> 24 hours): every 8 hours
	- continuous infusion of heparin (600 to 800 IU/hou via the arterial inflow cannula	

APTT = activated partial thromboplastin time, HIT = heparin-induced thrombocytopenia, iv = intravenous.

### Management

After insertion of the iLA-system, a de-escalation of invasive ventilatory variables (tidal volume, plateau pressure, frequency, F_i_O_2_) was performed, aimed at preventing the injured lung from further (ventilator-induced) damage.

Changes in ventilatory parameters were applied as follows: reduction of tidal volume (V_T _6 ml/kg ideal body weight (IBW) or lower), of inspiratory plateau pressure (p_plat _≤ 30 cmH_2_O) and of respiratory frequency (25 breaths/minute or less), adoption of PEEP following the 'high alveoli'-concept of the ARDS Clinical Trials Network [11]. Target arterial blood gases during iLA-treatment were: PaO_2 _70 mmHg or higher and pH 7.25 or higher.

After treatment of the underlying lung damage leading to a further reduction in invasive mechanical ventilation (F_i_O_2 _less than 0.5, PEEP 12 cmH_2_O or less, assisted spontaneous breathing) weaning from the iLA-system was initiated by starting a 'cessation trial' (reduction of iLA sweep oxygen gas flow to 1 L/minute) for a duration of two hours. If no major deterioration of gas exchange variables was observed and no significant increase in patients minute volume ventilation or tachypnoea (40 breaths/minute or more) occurred, the cannulae were manually removed, followed by sufficient and continuous compression of the insertion sites for at least 30 minutes. Thereafter, a pressure banding was applied for a period of 24 hours. Weaning from mechanical ventilation followed a clinical ICU-guideline, in which a spontaneous breathing trial (SBT) was implemented. In brief, SBT was daily screened and performed with certain pulmonary (FiO_2 _0.4 or less; PEEP 8 cmH_2_O or less, SaO_2 _90% or higher), haemodynamic, neurological and metabolic conditions fulfilled. SBT was carried out over one hour and the patient was extubated if no marked deterioration in gas exchange, haemodynamic and stress-associated parameters was observed.

The management of adverse events followed a consented written algorithm. In cases of severe and persistent ischaemia of a lower limb (no pulse oxymetry and doppler assessment of leg arteries for more than two hours and/or visual impression of impaired perfusion and/or acute elevation of ischaemia-indicating laboratory values (creatine kinase, lactate)) an immediate removal of the cannulae was prescribed.

### Statistical analysis

Statistical analysis was performed using SPSS Software, version 16.0 (SPSS Inc., Chicago, IL, USA). As revealed by the Kolmogorov-Smirnov method, most datasets significantly varied from the pattern expected if they were drawn from a population with a normal distribution. Non-parametric procedures were therefore applied for intergroup (Mann-Whitney Rank Sum Test, Kruskal-Wallis analysis of variance (ANOVA) on Ranks), and intragroup analysis (Wilcoxon Signed Rank Test, Friedman Repeated Measures ANOVA on Ranks). Results were considered significant at p < 0.05. Data are presented as median and interquartile range unless otherwise specified.

## Results

Initiation of iLA resulted in a significant improvement in arterial oxygenation and a marked removal in arterial carbon dioxide within two hours allowing a rapid reduction in F_i_O_2_, minute ventilation and inspiratory plateau pressure (Table 2). Furthermore, we were able to set lung protective tidal volume consequently to a level below 6 ml/kg IBW without provoking severe hypercapnia and/or acidosis. Following our PEEP/FiO_2 _trial, we found no changes in the PEEP level within 24 hours after starting iLA. Insertion of iLA did not induce haemodynamic instability although mean arterial pressure or the amount of continuous infusion of noradrenaline remained unchanged, or showed a tendency towards stabilisation. Sequential organ failure assessment (SOFA)-score did not change significantly after 24 hours following initiation of iLA.

**Table 2:** Changes in gas exchange, cardiovascular and respiratory variables before and during interventional lung assist (iLA) treatment

	**Pre-iLA**	**2 hours after insertion**	**24 hours after insertion**
PaO_2_/FiO_2_	75 (62 to 130)	102 (70 to 127) *	110 (86 to 160) *
PaCO_2 _(mmHg)	73 (61 to 86)	44 (36 to 54) **	41 (34 to 48) **
Arterial pH	7.23 (7.16 to 7.30)	7.38 (7.32 to 7.46) **	7.44 (7.37 to 7.49) **§
MAP (mmHg)	73 (65 to 80)	83 (75 to 91) **	81 (76 to 90)
Noradrenaline (μg/kg/minute)	0.16 (0.04 to 0.35)	0.11 (0.03 to 0.28)	0.09 (0.02 to 0.24) *
iLA-flow (L/minute)	-	1.8 (1.6 to 2.0)	1.7 (1.5 to 2.0)
FiO_2_	1 (0.8 to 1.0)	0.8 (0.7 to 1.0) **	0.7 (0.6 to 0.9) **§S
MV (L/minute)	11.5 (9.3 to 12.5)	8.6 (6.4 to 10.5) **	6.6 (5.5 to 8.3) **§S
V_T _ml/IBW	6.6 (5.3 to 7.2)	5.0 (4.0 to 6.4) **	4.4 (3.4 to 5.4) **§S
RR (breaths/minute)	25 (22 to 27)	23 (20 to 30)	21 (18 to 26)
P_plat _(cmH_2_O)	35 (31 to 38)	34 (30 to 37)	30 (26 to 34) **
PEEP (cmH_2_O)	17 (14 to 20)	15 (11 to 19) *	17 (14 to 20)

Variables are presented as median values (interquartile ranges).

* p < 0.05 in comparison with pre-iLA

** p < 0.01 in comparison with pre-iLA

§p < 0.05 in comparison with two hours after insertion

§S p < 0.01 in comparison with two hours after insertion

FiO_2 _= fraction of inspired oxygen; MAP = mean arterial pressure; MV = minute ventilation; PaCO_2 _= partial pressure of carbon dioxide in arterial blood; PaO_2 _= partial pressure of oxygen in arterial blood; P_plat _= plateau pressure; PEEP = positive end expiratory pressure; RR = respiratory rate; V_T _= tidal volume.

Twenty-six of 51 patients (50.9%) survived ARDS, non-survivors had a significant higher age, although the severity of disease (SOFA-score) and the severity of lung injury (Lung Injury Score) were not different between survivors and non-survivors (Table 3).

**Table 3:** Patients characteristics and outcome

	**All**	**Survivors**	**Non-survivors**
Patients	51 (100%)	26 (50.9%)	25 (49.1%)
Age (years)	52 (40 to 59)	44 (25 to 53)	58 (51 to 63) **
Female/male ratio	8/43	5/21	3/22
Body mass index	26.2 (23.7 to 31.1)	26.6 (23.8 to 31.1)	25.1 (23.9 to 28.4)
Days on ventilator (prior iLA)	4 (2 to 7)	3 (1 to 6)	5 (2 to 8)
Days on iLA	8 (6 to 11)	8 (6 to 10)	8 (4 to 16)
Lung injury score (Murray)	3.3 (3.25 to 3.7)	3.3 (3.3 to 3.7)	3.3 (3.1 to 3.7)
SOFA score (prior iLA)	10 (8 to 12)	9 (7.5 to 12)	10 (9 to 12)
SOFA score (24 hours after insertion)	10 (6 to 11)	9 (6 to 11)	10 (7 to 12)

Data are presented as median values (interquartile ranges), except female/male ratio.

** p < 0.01 in comparison with survivors.

ILA = interventional lung assist; SOFA = sequential organ failure assessment.

The frequency of complications is reviewed in Table 4. Transient episodes of lower limb ischaemia after arterial cannulation occurred in three patients. This resulted in removal of the cannula, resulting in normalisation of distal perfusion. Surgical intervention was mandatory in one patient who developed a compartment syndrome. From those four ischaemic complications, three patients had been cannulated with a 17 Fr cannula for the arterial approach. Other complications were rarely seen (cannula thrombosis, bleeding). In a total of six patients (11.9%) adverse events were observed. No adverse event had an effect on outcome.

**Table 4:** Frequency of complications

**Complication**	**Number of patients (%)**
Ischaemia of lower limb	3 (5.9)
Cannula thrombosis	1 (1.9)
Bleeding during cannulation	1 (1.9)
Compartmental syndrome (limb)	1 (1.9)
**All**	**6 (11.8)**

## Discussion

The main results of our prospective case series are: a change in the indication spectrum resulted in a trend toward an increased survival rate compared with the retrospective comparator study [4]; and a significant reduction in the incidence of adverse events, especially ischaemic complications. In our patients, iLA enabled a safe application of lung protective tidal volume (V_T _= 6 ml/kg IBW) and in some patients even less than 6 ml/kg without provoking severe acidosis. The combination of very low V_T _with high PEEP allowed the limitation of plateau pressure at 30 cmH_2_O or lower and, thus the avoidance of barotrauma. The use of smaller cannulae and an improved cannulation technique (careful assessment of the vessels by ultrasound, cowork-insertion by two experienced physicians) may facilitate the reported improvements. With smaller cannulae (arterial 17 Fr or lower), a sufficient blood flow of 1.0 to 1.5 L/minute allowed for adequate carbon dioxide-removal within the circuit [12]. Although the support of a strict lung protective ventilation [13] is our main goal for the use of iLA in ARDS patients, the insertion of smaller cannulae is sufficient to reach such a goal and minimises adverse ischaemic events. Improvements in cannulae allowed the use of shorter (9 cm versus 14 cm) and thinner (13 versus 15 Fr) cannulae for arterial cannulation. It is conceivable that further evolution of this technology will contribute to greater improvements in the risk-benefit ratio for the use of arteriovenous iLA.

Over the past three decades, an intensive scientific debate on the effectiveness and possible harm of extracorporeal lung assist systems was stimulated by clinical investigations studying ECMO or extracorporeal carbon dioxide removal in rescue situations (life-threatening hypoxaemia/hypercapnia) of patients with ARDS [14-17]. In small prospective randomised investigations, no clear survival benefit was demonstrated using ECMO in comparison to 'conventional' treatment, and the complication rate was shown to be high (more than 50%) [14]. From today's point of view there were two major limitations regarding the results and the interpretation of previous studies. Specifically, patients were ventilated in the mode of the 'Pre-ARDSNetwork-era' with high tidal volumes and relatively low PEEP, thus a harmful potential of ventilator-induced lung injury was postulated; and the 'historic' use of roller pumps might have aggravated the high complication rate (demand for elevated doses of heparin, induction of haemolysis) and in the past, the technique of miniaturised centrifugal pumps has been advocated [18-20].

A retrospective analysis of a new iLA system using an arteriovenous shunt and a membrane lung, characterised by an extremely-low flow resistance, demonstrated effective carbon dioxide removal and a moderate oxygenation improvement in severe ARDS, but no survival benefit in life-threatening hypoxaemia/hypercapnia was observed [4]. This was potentially because of the lack of a clear algorithm and indication strategy. Since 2004, we have worked to change the use of iLA in terms of defining indications, refining insertion technique and optimising the management of possible complications. In contrast to a previously described clinical concept aimed at 'rescue' in life-threatening gas exchange limitation, the new algorithm introduced in this report stipulates the insertion of iLA mainly for extracorporeal carbon dioxide-removal purposes facilitating lung-protective ventilation. In our view, the most important aspect of the presently described algorithm is the indication strategy. In summary, we withdrew the iLA-system from life-threatening 'rescue-situations' towards the support of lung-protective ventilation in acute lung injury or early ARDS on the threshold of becoming 'established' ARDS [10]. In this concept, patients with a critically impaired pulmonary gas exchange remain candidates for ECMO.

A comparison of cannula diameter, iLA effects on gas exchange and complications between the present study and our earlier work [4] demonstrated that the improved methodology described here led to a significant reduction in the rate and nature of complications. In the present prospective pilot study, six patients (11.8%) had complications because of iLA (transient ischaemia of a lower limb in three patients), while in our recent retrospective analysis the incidence of complications was 24.4%, and serious ischaemic complications occurred in nine patients (10%, p < 0.05). Furthermore, there is a trend toward a decrease in mortality in our prospective cohort (49.1%) compared with the retrospectively analysed patients suffering from ARDS (58.9%).

Nevertheless, our present analysis has some limitations. Having been conducted in a single-centre, the study might be subject to bias. Furthermore, the data stem from an expert team of intensivists and perfusionists, having a long 'learning curve' experience (> 150 applications) with the iLA system. Taking this into consideration, we present – in contrast to our previous analysis – clinical data resulting from a strict algorithm with defined indications (and contraindications).

Importantly. however, was also the identification of patient groups that do not presently receive benefit from our iLA algorithm. Specifically, in haemodynamically unstable patients requiring high doses of vasopressors (noradrenaline 0.4 μg/kg/minute or higher) or in patients with severe hypoxaemic ARDS, a pump-driven ECMO is still the rescue measure of choice.

Our new algorithm specifies indications for iLA differing from ECMO that underscore the respective differences in therapy concept. Although ECMO, characterised by high blood flow, resembles an 'artificial lung' by producing significant exchange of carbon dioxide and oxygen, iLA with its low blood flow provides impressive carbon dioxide elimination with a modest oxygenation improvement. Consequently iLA could be used as an adjunct to mechanical ventilation affording optimised lung-protective ventilation strategies, with the objective of giving the lungs time to heal [21].

## Conclusion

Our data demonstrate that iLA can be an important tool enabling advanced lung-protective ventilation in patients suffering from ARDS. The use of an indication algorithm for iLA in early ARDS, combined with a refined application technique was associated with efficient carbon dioxide removal and a reduction in the incidence of adverse events, especially ischaemia complications. With the ongoing technical evolution of smaller cannulae, more efficient gas exchange membranes and easy system handling, we hypothesise that iLA could serve as an extracorporeal assist to support respirator ventilation by enabling low tidal volume and reduced inspiratory plateau pressure as an important tool in ARDS management. This hypothesis is currently being tested by a prospective multicentre randomised trial (ClinicalTrials NCT 00538928).

## Key messages

• The algorithm introduced in this report stipulates the insertion of iLA mainly for extracorporeal carbon dioxide-removal purposes enabling lung protective ventilation strategies.

• iLA allowed a safe application of lung-protective tidal volume (6 ml/kg or less) and a reduction in inspiratory plateau pressure without provoking severe acidosis.

• The use of iLA in early ARDS, combined with a refined application technique including the use of size-adapted cannulae was associated with an efficient carbon dioxide removal and a low incidence of adverse events in this prospective pilot study.

## Abbreviations

ANOVA: analysis of variance; ARDS: acute respiratory distress syndrome; ECMO: extracorporeal membrane oxygenation; FIO_2_: fraction of inspired oxygen; Fr: French; IBW: ideal body weight; ILA: interventional lung assist; PaO_2_: partial pressure of oxygen in arterial blood; PEEP: positive end expiratory pressure; SBT: spontaneous breathing trial; SOFA: sequential organ failure assessment; V_T_: tidal volume.

## Competing interests

TB received lecture honorary from Novalung GmbH. The other authors declare that they have no competing interests.

## Authors' contributions

MZ made substantial contributions in data acquisition, patient care and writing the manuscript. TB contributed to the study design, statistical analysis and interpretation of data as well as final approval of the manuscript. MA, AP, LR and TM equally made substantial contributions in data acquisition and patient care as well as reviewing the manuscript. HS and BG critically revised the manuscript for important intellectual content.
